# Development and validation of a deprescribing tool relevant to older persons in India using modified Delphi consensus technique

**DOI:** 10.1186/s12877-025-06665-3

**Published:** 2025-12-02

**Authors:** Ratinder Jhaj, Atiya R. Faruqui, Santanu Kumar Tripathi, Minakshi Dhar, Rajnish Joshi, Bhushan Shah, Snehil Gupta, Rehan Ul-Haq, Danish Javed, Ajay Kumar Shukla, Shubham Atal, Anindo Majumdar, Jerin Jose Cherian, Sowparnika Treasa Sabu, Sandeep Tiwari, Bushra Siddiqui, Padmini Devi D, Ashish Kumar Kakkar, Sandhiya Selvarajan, Arunansu Talukdar, Vishakha Jain, Bidita Khandelwal, Ravi Kirti, Ashish Goel, Vinod Joseph Abraham, Barun Kumar, Satyajit Singh, Kiron Varghese, Tarun Goyal, Pankaj Kandwal, John Ashutosh Santoshi, Nishant Goyal, Palanimuthu Thangaraju Sivakumar, Mathew Varghese, Jaideep Menon, S. P. Dhaneria, Jyotirmoy Pal, Arvind Mathur, Sandeep Grover, Anil Jain

**Affiliations:** 1 Department of Pharmacology, AIIMS, Bhopal, Madhya Pradesh India; 2 Department of Pharmacology, St. John’s Medical College, Bengaluru, Karnataka India; 3 Jagannath Gupta Institute of Medical Sciences & Hospital, Budge Budge, Kolkata, West Bengal India; 4 Pharmacology, St. Johns Medical College, Bengaluru, Karnataka India; 5 Department of General Medicine, AIIMS, Bhopal, Madhya Pradesh India; 6 Department of Cardiology, AIIMS, Bhopal, Madhya Pradesh India; 7 Department of Psychiatry, AIIMS, Bhopal, Madhya Pradesh India; 8 Department of Orthopedics, AIIMS, Bhopal, Madhya Pradesh India; 9 Senior Medical Officer (AYUSH), AIIMS, Bhopal, Madhya Pradesh India; 10 Department of CFM, AIIMS, Bhopal, Madhya Pradesh India; 11 Clinical Studies and Trials Unit, Division of Development Research, Indian Council of Medical Research (ICMR), New Delhi, India; 12 Department of Global Public Health, Karolinska Institute, Stockholm, Sweden; 13 Division of Development Research ICMR Headquarter, New Delhi, India; 14 Pharmacology, St. Johns Medical College, Bengaluru, Karnataka India; 15 Department of Pharmacology, PGIMER, Chandigarh, India; 16 Department of Clinical Pharmacology, JIPMER, Pondicherry, India; 17 Geriatric Medicine Kolkata Medical College, West Bengal, India; 18 General Medicine, AIIMS, Bibinagar, Telangana India; 19 Department of General Medicine, SMIMS, SMU, Gangtok, Sikkim India; 20 Department of General Medicine, AIIMS, Patna, Bihar India; 21 Department of Internal Medicine, AIIMS, Mohali, Punjab India; 22 Department of Community Medicine, CMC Vellore, Tamil, Nadu India; 23 Deparment of Cardiology, AIIMS, Rishikesh, Uttarakhand India; 24 Department of Cardiology, AIIMS, Raipur, India; 25 Department of Cardiology, St. John’s Medical College Hospital, Bengaluru, Karnataka India; 26 Department of Orthopedics, AIIMS, Bhatinda, Punjab India; 27 Department of Orthopedics, AIIMS, Rishikesh, Uttarakhand India; 28 Department of Psychiatry, CIP Ranchi, Jharkhand, India; 29 Department of Psychiatry, National Institute of Mental Health and Neurosciences, Bengaluru, Karnataka India; 30 St. John’s Medical College Hospital, Bengaluru, Karnataka India; 31 Department of Cardiology, Amrita Institute of Medical Sciences, Kochi, Kerala India; 32 Pharmacology, RG Gardi Medical College, Ujjain, Madhya Pradesh India; 33 Department of General Medicine, R G Kar Medical College & Hospital, Kolkata, West Bengal India; 34 Geriatric Medicine, Director and Managing Trustee, ACMERI, Jodhpur, Rajasthan India; 35 Department of Psychiatry, PGIMER, Chandigarh, India; 36 Indian Spine Journal, Annals of National Academy of Medical Sciences, Delhi, India

**Keywords:** Deprescribing, Tool, Elderly, Old, Indian, Potentially inappropriate medications

## Abstract

**Background:**

Polypharmacy is common in older persons, and some of the prescribed drugs may be potentially inappropriate medications (PIMs), whose risks outweigh their benefits. It is therefore necessary to review medication intake of older patients and deprescribe the ones that are not appropriate. Although there are many tools for deprescribing, none of these have been developed for the Indian context, where there is widespread use of fixed dose combinations (FDCs), medicines from different systems of medicines, and different levels of healthcare. Also, some of the medications listed in existing tools are not marketed in India, while some which are commonly prescribed in the country are not listed in the tools. We therefore undertook to develop a deprescribing tool for the older persons in India.

**Methods:**

A draft deprescribing tool was developed through a modified Delphi consensus process by 18 panellists. Item level content validity index (I-CVI) and scale level content validity index (S-CVI) were calculated from the Delphi round 1 and 2 responses. In addition, face and content validity of the tool was carried out by 6 experts and content validity ratio (CVR’) and I-CVI were calculated.

**Results:**

Our draft Deprescribing tool included 63 drugs/groups organized in three sections-drugs/drug groups to avoid generally in all older patients (39 items), herb-drug interactions (5 interactions) and drugs which should be avoided in renal dysfunction (15 allopathic and 4 herbal medicines). After Delphi process and tool validation, ten drugs/drug groups were removed due to CVR’ value < 0.62 or I-CVI value < 0.67, and recommendations and/or alternatives were modified for nine drugs/groups, so that the final the tool includes 52 items – 30 drugs/drug groups to be avoided in all older persons, 7 herb-drug interactions and 15 drugs to be avoided in renal dysfunction. The scale-level content validity index (S-CVI) was 0.87 for the entire tool, which is higher than the generally acceptable 0.8.

**Conclusion:**

A deprescribing tool containing drugs/drug groups commonly prescribed and relevant for the older persons in India, including important herb-drug interactions, and restricted to drugs available and prescribed in India, has been developed through the Delphi process followed by tool validation.

**Supplementary Information:**

The online version contains supplementary material available at 10.1186/s12877-025-06665-3.

## Background

Older persons often suffer from multiple chronic illnesses, requiring medications for treatment and prophylaxis [1]. They are thus frequent candidates for polypharmacy, with estimates of proportion of persons over 65 years receiving polypharmacy varying between 30% and 50% globally [2–6] in ambulatory patients, and reaching as high as 91% in older residents of care homes [7]. Indian studies too have reported prevalence of polypharmacy to be 49% to 66.2% among those aged 60 years or more [8–10]. Polypharmacy, most commonly defined as prescription of five or more drugs [11], is associated with increased risk of serious adverse events, including falls, cognitive impairment, functional decline, hospitalization, increased length of hospital stays and death [2, 3, 12]. Polypharmacy-related adverse drug events have been reported to occur in 25% of ambulatory care patients [13]. Moreover, adverse drug events can be misinterpreted as new disorders, leading to prescriptions for new medications, or a prescribing cascade [14]. Use of medications not clinically needed also adds to unnecessary drug costs, particularly relevant to a country like India where households spend most of their out of pocket healthcare expenditure on medications [15].

Although polypharmacy in itself is not equivalent to inappropriate drug use, many studies [14, 16, 17] including those from India [8, 9], have highlighted the use of potentially inappropriate medications (PIMs) in older patients. PIM is defined as a medication whose adverse effects in older adults may outweigh its clinical advantages [8, 18]. Globally, prevalence of PIMs in prescriptions of older patients has been reported to vary from 11.5% to 62.5% in ambulatory older persons [2, 5, 19] to as many as 85.2% in case of older patients on long term care [7, 20]. Bhagavathula et al., in their systematic review and meta-analysis of 27 studies evaluating polypharmacy in India, reported PIMs in older persons among different regions varying from 17 to 33%, with a pooled prevalence of 28% [8]. The prevalence of PIM use also varied between inpatient (31%) and outpatient (25%) settings, and was lower in government hospitals (25%) compared to private ones (31%).

Deprescribing, a process of tapering, stopping, discontinuing, or withdrawing drugs, with the goal of managing polypharmacy and improving outcomes [21], can reduce use of inappropriate and unnecessary medications, improve quality of life and reduce frailty, adverse events, drug interactions, hospitalizations, medication costs and costs of morbidity and hospitalisations [2, 16, 22, 23].

There are many tools for deprescribing, including implicit and explicit tools [24]. Implicit tools, like the Medication Appropriateness Index (MAI) [25], are context-dependent and require an individualized approach that relies on the evaluator’s expertise and knowledge, and are time consuming, while explicit tools are generally lists of medications or criteria which can be applied with little or no clinical judgement and are less time consuming [26]. Explicit deprescribing tools include American Geriatrics Society 2023 Updated AGS Beers Criteria^®^ for Potentially Inappropriate Medication Use in Older Adults [27] and Screening Tool for Older Person’s Prescriptions (STOPP) version 3, 2023 [28].

However, none of these tools have been developed for the Indian context. We therefore used the available deprescribing tools to develop a deprescribing tool for older persons in India, aged 60 years or more. Incidentally, there is no single cut-off for classification of older person/elderly [29]. Many countries consider a person 65 years or more as elderly/older person, and most tools for deprescribing mention this cut-off age. However, according to the United Nations, and in India, persons more than 60 years are considered as older/elderly [30].

## Methodology

The study was a collaborative work conducted at All India Institute of Medical Sciences (AIIMS), Bhopal, India and St. John’s Medical College, Bengaluru, India. Modified Delphi Technique was used as it allows for repetitive anonymous voting by a large number of experts, allowing for inclusion of experts with wide specialty and geographical spread, combined with a final in-person meeting [31]. Other consensus development methods (CDMs) presented limitations such small number of participants (Nominal Group Technique and Consensus Development Conference, usually involving up to 10 participants). The RAND/UCLA Appropriateness Method (RAM), is useful to decide appropriateness from a list of predetermined items, and therefore was not suitable for development of a new tool [32]. ACCORD (ACcurate COnsensus Reporting Document) guideline for consensus methods in biomedicine [33] was used to report the results.

### Step 1: item generation

Review of existing deprescribing interventions was carried out by members of the Steering Committee, RJ, SA, AS (AIIMS Bhopal) and AF (St. John’s Medical College, Bengaluru) to prepare the first draft of the deprescribing tool. A comprehensive search was conducted across multiple electronic databases, including PubMed, Web of Science, and Embase, including articles published up to December 2023, using the Medical Subject Headings (MeSH) terms “deprescribing,” “elderly,” “older,” “polypharmacy”. Non-English sources were included if English translation were available. However, since we wanted to restrict to published deprescribing tools, gray literature was excluded. Article titles and abstracts were screened, followed by screening the full text of all identified papers. A multidisciplinary core expert group of six members including one specialist each from General Medicine, Geriatric Medicine, Cardiology, Psychiatry, Orthopedics and Ayurveda (Supplementary Table 1) reviewed the draft tool.

### Step 2: tool development through three step modified delphi consensus method

Two rounds of web-based survey (Survey Monkey^®^) and a final online meeting- was conducted for content validity of the deprescribing tool, as used by various researchers for the development of a number of the existing explicit deprescribing tools [34].

### Selection of panelists

A total of 18 specialists (Supplementary Table 1), three each from the above fields, from various tertiary healthcare institutes across the country, formed the Delphi panel. To ensure representation from across the country, a list of experts with experience in each of the concerned specialties, working in tertiary healthcare institutes in different regions of the country was prepared by accessing the institute websites. Three experts per specialty were invited via e-mail to participated as Delphi panelists, till requisite number had consented to participate.

### Round 1

The draft tool was circulated through Survey Monkey^®^ to all 18 panel members accompanied by a clear explanation of the objectives of the study and specific instructions for member participation. Each expert was asked to vote by marking each drug on a 5-point Likert scale where 5 = strongly agree, 4 = agree, 3 = neutral, 2 = disagree, 1 = strongly disagree. Experts were also given the opportunity to provide comments and suggest additional drugs that may not have been included when developing the initial list. For each drug, consensus was based on the median Likert response and interquartile range. A 70% to 80% agreement level is generally considered to be suitable for consensus building through the Delphi process [35]. Also, Lavan et al. [36], who developed the STOPPfrail deprescribing tool for older persons with frailty, suggested that at least 75% of experts must agree on an item in order to achieve content validity when there are at least 10 experts participating in consensus development. Thus, a median value of 4 or 5 with a 25th centile of ≥ 4 was accepted for inclusion in the tool, i.e. only drugs/groups with at least 75% of respondents agreeing or strongly agreeing (i.e., agreement among 14 of 18 experts) were included. Drugs not meeting 75% agreement were redistributed to the panelists for round 2.

### Round 2

The list of drugs that did not meet consensus from round 1 was resent to all 18 members. In round 2, the experts used the same rating method as described for round 1, but with the knowledge of the group comments. Final responses were analyzed as described for round 1, and drugs not meeting 75% agreement were retained for discussion in round 3.

### Round 3

Round 3 was conducted using an online meeting platform. Panel members were encouraged to discuss the remaining drugs (inconclusive during rounds one and two), until agreement was reached to retain, modify, or eliminate a drug from the final deprescribing tool. 75% agreement was still used to determine acceptance or rejection of a drug. Round 3 consensus was obtained by voting using a show of hands and anonymity was not retained.

### Tool validation

I-CVI and S-CVI were calculated from the Delphi round 1 and 2 responses as described by Almanasreh et al. [37]. In addition, face and content validity of the tool developed through the modified Delphi method was established by six experts (one each from the Clinical Pharmacology, General Medicine, Geriatric Medicine, Cardiology, Psychiatry and Orthopedics). Experts were asked to rate the tool through Survey Monkey ^®^. Section 1, that is, drugs to be avoided in the older persons were rated on 3 aspects-

- Need for the drug/group to be included in the deprescribing tool. This was done by rating each item as either of the following three responses: not necessary, useful but not essential or essential.
- Relevance of the recommendations, suggested alternatives and rationale. These were rated on a 4-point scale as one of the following scores: 1[not relevant], 2[somewhat relevant], 3[quite relevant], 4[very relevant].
- Clarity of the recommendations, suggested alternatives and rationale. These were rated on a 4-point scale as one of the following scores:1[not clear], 2[somewhat clear], 3[quite clear], 4[very clear].

Experts were also able to add any remarks in the space provided to them.

Section II - Herb-drug-interactions and section III-Drugs to be avoided in renal dysfunction, were rated on one aspect only, that is, need for the drug/group to be included in the deprescribing tool. This was done by rating each item as either not necessary, useful but not essential or essential. Relevance, clarity of the recommendations and suggested alternatives and rationale were not rated for these two sections, since there were no alternatives suggested and recommendations only included avoiding the drugs in older persons.

Content validity ratio (CVR’) was obtained for each item by measuring the proportion of essential agreements in relation to the total number of experts, with an acceptable value of ≥ 0.67, in accordance with the modified Lawshe Table [38]. Item level content validity index (I-CVI) was calculated as the number of experts giving a rating 3 or 4 to the relevance and clarity of each item, divided by the total number of experts [39].

## Results

The draft deprescribing tool contained 63 drugs/groups organized in 3 Sections (Supplementary Table 1), i.e. Section I: This contained 39 drugs/drug groups divided into 7 Therapeutic Groups which should be avoided in older persons. Section II: This included 5 Herb-Drug Interactions involving herbal medicines and allopathic drugs commonly used in older persons. Section III: Included 15 allopathic and 4 herbal medicines to be avoided in Renal Dysfunction.

### Delphi round 1

In round 1, there was < 75% agreement for only 5 drugs/groups. Four of these were herbal medicines and one group were the nonbenzodiazepine (non-BZD) receptor agonist hypnotics (Z-drugs). These were re-sent in round 2, along with suggested modifications in recommendations.

### Delphi round 2

There was > 75% agreement to 2 of the 5 drugs circulated for round 2. Modification in recommendations for non-BZD receptor agonist hypnotics and additional information for herb-drug interaction with furosemide and licorice were agreed to by > 75% of experts. The remaining three drugs, that is licorice, noni juice and ephedra alkaloids (ma huang) in renal dysfunction still did not reach the required level of agreement.

### Delphi round 3

The third and final round was conducted in the form of an online meeting between the Delphi panellists and study investigators. Twelve of the 18 Delphi panellists attended the meeting, vote of the remaining members was taken via e-mail. The results of round 2, and the modifications made thereafter were discussed. It was decided to remove herbal medicines from section III (drugs to be avoided in renal dysfunction), since these are not prescribed by practitioners of modern medicine, hence dose adjustments or deprescribing in renal dysfunction is not applicable. However, section II, that is commonly used herbal medicines with moderate level of drug interaction with allopathic medicines was retained.

Of the four herbals in section III, Ginkgo biloba was already included in section II. While the pharmacokinetic interaction of licorice with corticosteroids and other drugs due to inhibition of the CYP3A4 isozyme was already included as a herb-drug interaction, the pharmacodynamic interaction between licorice and antihypertensives, including diuretics, leading to a risk of sodium and water retention, hypokalemia, hypertension, particularly in patients with renal disease [40, 41] was added. Noni juice may increase risk of hyperkalemia with potassium sparing diuretics, angiotensinogen converting enzyme inhibitors (ACEIs), angiotensin II receptor blockers (ARBs), and beta-adrenergic blockers, and was hence moved to section II. Since ephedra alkaloids (ma huang) is not commonly used in India, it was decided to remove it from the Tool. Thus, after round 3, there were seven instead of five herb-drug interactions included.

In addition to the above changes, nine drugs which did have ≥ 75% agreement, but which received 2 or more similar comments from experts, and/or for which new evidence affecting the recommendations, alternatives, rationale or usage in India, was identified, were taken up for discussion. Five changes were agreed to by the majority of experts during the meeting. These included-

1. Tricyclic Antidepressants with potent anticholinergic action: Desipramine and doxepin were removed from PIM list and alternatives, respectively, since they are rarely used in the Indian setting.
2. Antipsychotic or anti-schizophrenia drugs (ASDs) with potent anticholinergic action: Olanzapine and clozapine may have to be used in patients not responding to other drugs or at risk of extra-pyramidal symptoms (EPS).
3. Peripheral alpha-1 blockers: Tamsulosin was moved to drugs to avoid instead of being included as an alternative, since a recent observational study shows association of tamsulosin with dementia in older men with BPH [42].
4. It was agreed to make the recommendations for insulin simpler.
5. Growth hormone was removed from Tool since it is rarely used in the Indian setting.

For four drugs/groups, the need to further look up references before a consensus could be arrived at was felt. The proposed changes were circulated to all experts and incorporated in the final draft of the tool. These included-

1. Modifications in alternatives for BZD and non-BZD sedative hypnotics. Melatonin, up to 2 mg, one hour before bedtime, used for the shortest duration necessary, was added to the alternatives for BZD and non-BZD sedative hypnotics, based on several studies [43, 44].
2. Cut-off for prescription of levothyroxine for subclinical hypothyroidism, was raised from 7 to 10 mIU/L, based on studies by Panday et al. [45] and Ross, D [46] as well as STOPP criteria 2023 [28].
3. In view of new evidence to support their use in older persons including >75 years and frail [47, 48], it was decided to remove statins from the Tool, that is consider them appropriate for primary cardiovascular prevention in older persons. It was also decided to further review the use of statins based on results of ongoing STAREE trial in Australia and PREVENTABLE trial in the US, both primary prevention trials of atorvastatin vs. placebo for adults ≥ and 75 respectively [49].
4. It was agreed to modify alternatives to gastrointestinal anticholinergics in diarrhea. Since medication may vary depending on cause of diarrhea, specific alternatives have been removed from the tool.

The above changes were incorporated in the deprescribing tool after Delphi round 3. The Deprescribing tool after round 3 included 59 drugs/drug groups, that is 37 drugs/drug groups which should be avoided in older persons, 7 herb-drug interactions and 15 drugs to be avoided in renal dysfunction. A QR Code with link to drugs.com was also included in the deprescribing tool for further information about drug interactions and drugs to be avoided in renal dysfunction.

### Content validation

**Fig. 1:**
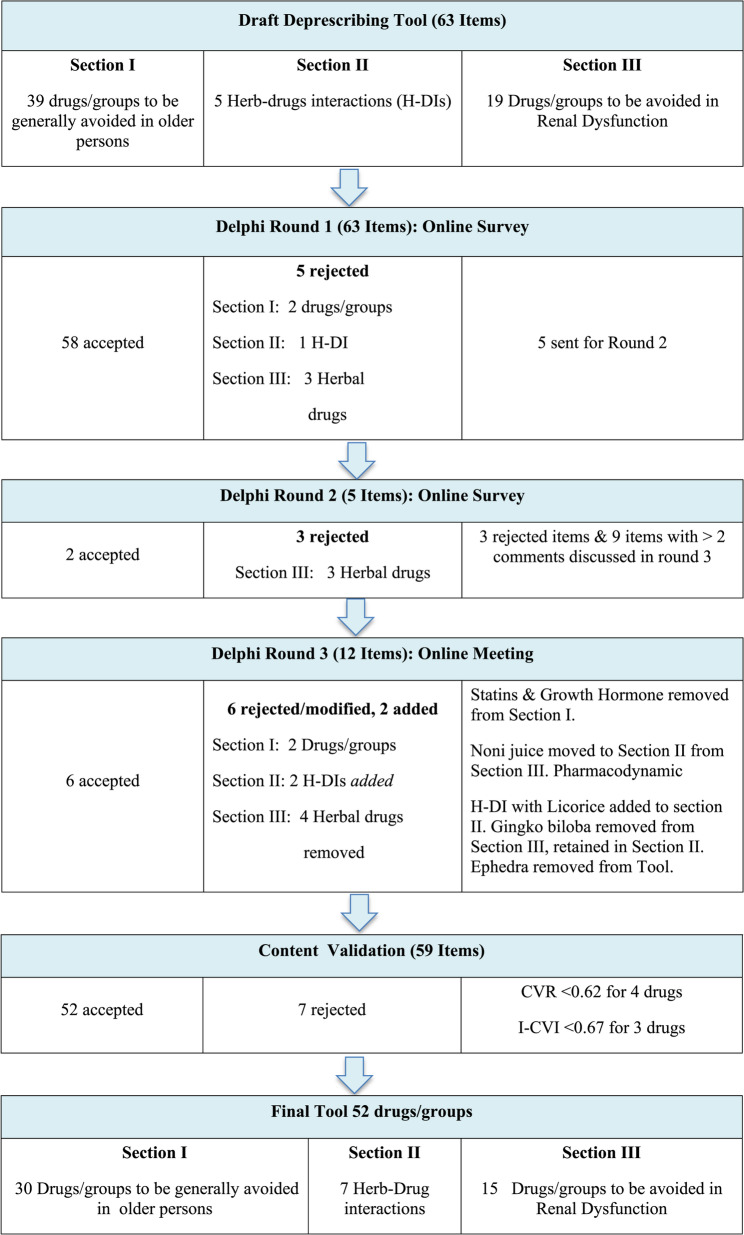
Flow chart summarizing the tool development process

During content validation, content validity ratio (CVR’) was less than 0.62 (0.5) for rivaroxaban, serratiopeptidase, insulin and megestrol (Table 1), which were therefore removed from the deprescribing tool. CVR’ for the remaining drugs/drug groups were between 0.67 and 1. Item level content validity indices (I-CVI) for relevance were between 0.67 and 0.82 for 16 of the drugs in section I and these were revised (Supplementary Table 1). I-CVI was less than 0.67 (0.5) for three drugs/drug groups, that is, all antipsychotic drugs (ASDs), amiodarone and dronedarone, which were removed from the tool. For the remaining drugs, the I-CVI were greater than 0.83. I-CVI for clarity for the drugs in section I were < 0.67 for 5 drugs and 0.67 for 12 drugs. These were reviewed and revised to improve clarity. The remaining drugs in section I had an I-CVI for clarity between 0.83 and 1. The final deprescribing tool after validation thus has 52 drug/drug groups. Which include − 30 drugs/drug groups which should be avoided in older persons, 7 herb-drug interactions and 15 drugs to be avoided in renal dysfunction. The scale-level content validity index (S-CVI) for the entire tool was 0.87 for the entire tool, which is higher than the generally acceptable 0.8. Figure 1 summarises the tool development process.

**Table 1:** Item- wise content validity scores of deprescribing tool

S No.	Drug/Drug Group	Delphi Process* N = 18		Content Validation *N* = 6					
		Total strongly agree & agree (A)	I-CVI A/18	Total essential & useful but not essential (B)	CVR’ A/6	Relevance		Clarity	
						Total quite relevant & very relevant (C)	I-CVI C/6	Total quite clear & very clear (D)	I-CVI D/6
Section I: Drugs to be generally avoided in the older persons (≥ 60 years)									
Group A: Drugs with Anticholinergic action general statement									
1.	Tricyclic antidepressants with potent anticholinergic action	17	0.94	5	0.83	5	0.83	5	0.83
2.	Antipsychotic drugs (ASDs) with potent anticholinergic action	15	0.83	5	0.83	4	0.67	4	0.67
3.	Skeletal muscle relaxants	16	0.89	6	1	6	1	6	1
4.	Anticholinergic antiparkinsonian agents	14	0.78	5	0.83	4	0.67	3	0.5
5.	Antimuscarinics for urinary incontinence	15	0.83	5	0.83	5	0.83	5	0.83
6.	Gastrointestinal antispasmodics and anticholinergics	17	0.94	5	0.83	5	0.83	5	0.83
7.	First-generation antihistamines	17	0.94	6	1	6	1	6	1
Group B: Sedatives & Hypnotics General Statement									
8.	Barbiturates: phenobarbital	18	1	6	1	6	1	6	1
9.	Benzodiazepines (BZDs)	18	1	5	0.83	4	0.67	4	0.67
10.	Nonbenzodiazepine BZD receptor agonist hypnotics (Z-drugs)	16	0.89	4	0.67	3	0.67	3	0.67
Group C: Drugs acting on the Cardiovascular System & Blood									
11.	Aspirin	16	0.89	5	0.83	4	0.67	4	0.67
12.	Warfarin	17	0.94	5	0.83	5	0.83	3	0.50
13.	Disopyramide	16	0.89	5	0.83	4	0.67	4	0.67
14.	Centrally acting alpha 2 agonists: Clonidine, Moxonidine	18	1	5	0.83	4	0.67	4	0.67
15.	Digoxin	18	1	5	0.83	4	0.67	4	0.67
16.	Non- dihydropyridine CCBs: Diltiazem, Verapamil	17	0.94	5	0.83	4	0.67	5	0.83
17.	Peripheral alpha-1 blockers	16	0.89	6	1	6	1	5	0.83
Group D: Analgesics and Non- Steroidal Anti-inflammatory Drugs (NSAIDs)									
18.	All Opioids	18	1	6	1	6	1	6	1
19.	Pethidine (Meperidine)	15	0.83	6	1	5	0.83	5	0.83
20.	All NSAIDs	15	0.83	6	1	4	0.67	4	0.67
21.	Indomethacin, Ketorolac	17	0.94	6	1	5	0.83	5	0.83
22.	Non-COX-2-selective NSAIDs	17	0.94	6	1	5	0.83	5	0.83
Group E-G: Gastrointestinal, Genitourinary and Endocrine drugs									
23.	Proton-pump inhibitors	17	0.94	5	0.83	4	0.67	2	0.33
24.	Metoclopramide	18	1	5	0.83	4	0.67	4	0.67
25.	Domperidone (alone or in combination with PPIs)	17	0.94	5	0.83	4	0.67	4	0.67
26.	Desmopressin	15	0.83	5	0.83	5	0.83	4	0.67
27.	Sulfonylureas (SUs)	16	0.89	5	0.83	4	0.67	4	0.67
28.	Pioglitazone	17	0.94	5	0.83	5	0.83	5	0.83
29.	Systemic estrogens for hormone replacement therapy	17	0.94	5	0.83	4	0.67	4	0.67
30.	Levothyroxine	16	0.89	5	0.83	4	0.67	3	0.5
Section II: Herb- Drug Interactions									
31.	Ginkgo biloba, Allium sativum with warfarin, aspirin, NSAIDs, dipyridamole, clopidogrel, prasugrel, ticagrelor	15	0.83	5	0.83	-	-	-	-
32.	*Panax ginseng* (Asian/Korean ginseng) with warfarin	15	0.83	4	0.67	-	-	-	-
33.	*Panax ginseng* (Asian/Korean ginseng) with insulin, sulfonylureas, glinides	14	0.78	4	0.67	-	-	-	-
34.	*Panax ginseng* (Asian/Korean ginseng) with furosemide	14	0.78	4	0.67	-	-	-	-
35.	Noni juice with potassium sparing diuretics, ACEIs, ARBs, beta adrenergic blockers	-	-	4	0.67	-	-	-	-
36.	Licorice (*Glycyrrhiza glabra)*/Mulaithi with corticosteroids & various drugs	14	0.78	4	0.67	-	-	-	-
37.	Licorice (*Glycyrrhiza glabra)*/Mulaithi with antihypertensives, including diuretics	-	-	4	0.67	-	-	-	-
Section III: Drugs which should be avoided in renal dysfunction									
38.	Anticoagulants: Dabigatran, Fondaparinux	16	0.89	5	0.83	-	-	-	-
39.	Anticoagulants (Factor Xa inhibitors): apixaban, edoxaban, rivaroxaban	15	0.83	5	0.83	-	-	-	-
40.	Bisphosphonates ^†^	16	0.89	5	0.83	-	-	-	-
41.	Digoxin	18	1	5	0.83	-	-	-	-
42.	Diuretics (K sparing) ‡	17	0.94	5	0.83	-	-	-	-
43.	Duloxetine	14	0.78	5	0.83	-	-	-	-
44.	Dofetilide	16	0.89	5	0.83	-	-	-	-
45.	Metformin	18	1	5	0.83	-	-	-	-
46.	Methotrexate	17	0.94	5	0.83	-	-	-	-
47.	NSAIDs	18	1	5	0.83	-	-	-	-
48.	Pethidine (Meperidine)	16	0.89	5	0.83	-	-	-	-
49.	Probenecid	16	0.89	5	0.83	-	-	-	-
50.	SGLT2 inhibitors^§^	15	0.83	5	0.83	-	-	-	-
51.	Tramadol	16	0.89	5	0.83	-	-	-	-
52.	Trimethoprim sulfamethoxazole	17	0.94	5	0.83	-	-	-	-

* Final Scores After Round 3

^†^Bisphosphonates: alendronate, ibandronate, risedronate, zoledronate etc.

‡Diuretics (K sparing): amiloride, triamterene spironolactone, eplerenone

^§^SGLT2 inhibitors: canagliflozin, dapagliflozin, empagliflozin etc

## Discussion

Chang et al. in 2011, compared the practicability of six different potentially inappropriate medication (PIM) criteria in geriatric outpatients with polypharmacy in Taiwan and concluded that the prevalence of PIMs varied significantly when different criteria were applied and advised caution in applying PIM criteria developed in other regions when medication availability in the local market is limited [50]. We therefore included only the drugs which are approved by the Central Drugs Standard Control Organization (CDSCO) and available in the Indian market in our tool. Although there is widespread use of FDCs in India [51], including those containing vitamin and mineral preparations [52], we included general guidance regarding avoidance of irrational FDCs in the tool, since the number of FDCs available is too large to include all.

Our draft Deprescribing tool included 63 drugs/groups organized in three sections. In round 1, there was 75% or more agreement for only 58 drugs/groups. In case of non-BZD receptor agonist hypnotics, there was less than 75% agreement with the recommendation, that is to avoid them in all older patients. The recommendations were therefore changed from ‘Avoid’ in the first draft to ‘Avoid, if possible, especially over 70 years. If necessary, use the lowest dose (up to half of the usual dose) and avoid for more than 2 weeks. In case of chronic insomnia may use up to 4–6 weeks, then taper and stop’ after round 2. This was based on studies, including a Cochrane systematic review suggesting that while Z-drugs may be safe for treatment of insomnia in older adults, they may lead to adverse effects, including falls and fractures when used for longer periods [28, 53]. Using half the usual dose is suggested by the EU [7]-PIM list [26]. In addition, melatonin, up to 2 mg, one hour before bedtime, used for the shortest duration necessary, was added as an alternative for BZD as well as non- BZD sedative hypnotics based on several studies [43, 44]. For BZD sedative hypnotics, classification into long- and short acting BZDs was removed since most literature recommends avoiding all of them (EU-PIM, STOPP), and recommendations were simplified in the final tool.

During tool validation, while antipsychotic drugs (ASDs) with potent anticholinergic action (ACA) had a CVR’ as well as I-CVI 0.83, the group ‘All ASDs’ had a CVR’ 0.83 but the I-CVI was only 0.67. The experts felt that the concern about anticholinergic action of ASDs was already addressed in the ‘ASDs with potent ACA’ group, and that all ASDs are not always inappropriate in all older patients. The 2023 Beers Criteria recommend avoiding BZDs only in conditions for which their use is *not* FDA-approved. Hence it was decided to remove ‘all ASDs’ as a separate group and include the caution about prolonged use under ASDs with potent anticholinergic action.

While practitioners of modern or allopathic medicine do not prescribe medicines from complementary systems, they need to be aware of major herbals with moderate level of drug interaction with modern medicines since these could affect the actions of drugs prescribed by them. We therefore included herbal medicines used in Ayurveda, for which there is evidence of clinically significant interactions with several modern medicines. These include Ginkgo biloba, Allium sativum (garlic, bulb/clove) *Panax ginseng* (Asian/Korean ginseng), Noni juice and Licorice (*Glycyrrhiza glabra)*/Mulaithi. While Ginkgo biloba and garlic increase risk of bleeding due to synergism with warfarin and anti-platelet agents [54–56], *Panax ginseng* decreases the anticoagulant effect of warfarin effect due to induction of cytochrome enzymes including CYP2C9 [56–58]. Ginseng also interferes with the diuretic effect of furosemide, due to an unknown mechanism [59]. On the other hand, it can exacerbate hypoglycemia with insulin, sulfonylureas and the glinides, which is of particular concern in older persons [60–63]. The mechanism of hypoglycemia with ginseng is not fully established, with both enhanced insulin sensitization and insulin secretion being proposed mechanisms [64]. Noni juice contains Noni Extract (*Morindo Citrifolia*), sometimes in combination with *Garcinia Combojia* and other herbal medicines. The high potassium content in Noni juice increases risk of hyperkalemia may increase risk of hyperkalemia with potassium sparing diuretics, angiotensinogen converting enzyme inhibitors (ACEIs), angiotensin II receptor blockers (ARBs) and beta-adrenergic blockers, particularly in patients with renal dysfunction [58, 65]. *Garcinia Combojia* may lead to liver injury, with possibility of drug interaction with other drugs causing liver toxicity [59]. Licorice inhibits Cytochrome P450 enzymes affecting metabolism of a number of drugs, including corticosteroids. Since ephedra alkaloids (Ma Huang) is not commonly used in India, it was decided to remove it from the tool.

Rivaroxaban, although agreed upon by more than 75% of the Delphi panellists, did not receive the required 0.62 agreement during tool validation. The experts were in agreement with Cohen et al., that the AGS 2023 recommendation to avoid rivaroxaban in favour of apixaban for long-term use is not adequately substantiated by the non-randomized studies. Many of these studies have very short follow-up periods, and many do not clearly articulate the balance between efficacy and risk. Further, many of these studies cannot account for the confounding role of frequent prescribing of inappropriate reduced dosing of DOACs [65]. Hence it was agreed that rivaroxaban should be used with caution and not avoided, and since we have included only drugs to be avoided in our tool, it was decided to remove it. Experts also felt that amiodarone should not be listed as a PIM for all older persons, as it may be actually be reasonable first-line therapy in patients with concomitant heart failure or substantial left ventricular hypertrophy if rhythm control is preferred over rate control [27]. Although several lists of PIMs for older persons do include amiodarone, the PRISCUS 2.0 includes it as an alternative to long-term propafenone [66]. Similarly, dronedarone needs to be avoided only in individuals with permanent atrial fibrillation or severe or recently decompensated heart failure and used caution in patients with heart failure with reduced ejection fraction (HFrEF) with less severe symptoms, that is NYHA class I or II [27], and is not included in most other lists of PIMs for older persons. Hence both these drugs were removed from the tool. For clonidine and moxonidine, as well as diltiazem and verapamil, the class name was added to indicate class effect and increase clarity.

Among other CNS drugs, after Delphi round 3 the recommendations for antipsychotic drugs (ASDs) with potent anticholinergic action (ACA) were modified to include that olanzapine and clozapine may have to be used as antipsychotic drugs (ASDs) despite potent anticholinergic action, in patients not responding to other drugs or in those with or at risk of developing extra-pyramidal symptoms (EPS) with the typical ASDs [25, 51, 52]. Among analgesics, recommendations for pethidine and NSAIDs were explained more clearly. For endocrine drugs, during tool validation, CVR’ for insulin was only 0.5, hence insulin was removed from the tool. Megestrol is rarely prescribed for weight gain in older persons and thus received a low CVR’ and was removed from the tool. The thyroid stimulating hormone (TSH) cut -off for prescription of levothyroxine for subclinical hypothyroidism, was raised from 7 to 10 mIU/L, based on studies by Panday et al. [45] and Ross, D [46] as well as STOPP criteria 2023 [28].

Thus, after three rounds of Delphi and further tool validation, 13 drugs/drug groups to be avoided in older persons were removed, including four herbal drugs to be avoided in renal disease. On the other hand, two herb-drug interactions were added and recommendations and/or alternatives were modified for another nine drugs/groups. As a result, our final deprescribing tool includes 52 drugs − 30 drugs/drug groups to be avoided in all older persons, 7 herb-drug interactions to look out for, and 15 drugs to be avoided in renal dysfunction.

To keep the tool practical and handy to use, we have only included drugs which should be generally *avoided* in older persons. Drugs which should be used cautiously in older persons have not been included as this makes the tool too exhaustive and, in any case, we believe all drugs should be used cautiously in older persons. Similarly, many drugs need dose adjustments in renal dysfunction, but we have included only drugs which need to be avoided at least at some threshold of creatinine clearance/eGFR. On the same lines, there are innumerable potential drug-drug interactions, but a complete list would make the tool too extensive and there are already existing online resources for checking drug interactions. We have therefore included a QR code with a link to Drugs.com where all drug-drug interactions as well as dose adjustments for renal disease can be found free of cost. We only included 7 clinically important herb -drug interactions as these herbal medicines are often taken by older persons in India and the tool will serve as a reminder for the physicians to ask for a history of medication with these and any other relevant traditional and complementary medicines.

### Limitations of the study

Geriatrics is a field in evolution in India, where most healthcare institutes do not have a separate department of geriatrics. Older patients are hence cared for by several specialties. We therefore included experts from the most relevant specialties, including General Medicine, Geriatric Medicine, Cardiology, Psychiatry, Orthopedics and Ayurveda, for the development of this tool. This meant that not all experts were equally familiar with all therapeutic classes of drugs. However, we did not give any weightage to responses by experts for drugs particularly prescribed in their specific field, as this would have complicated the process. All responses were treated as equal, which is a limitation of the work. Also, despite the rigorous methodology of modified Delphi, some degree of subjectivity in expert consensus cannot be ruled. Lack of real-world implementation and evidence of effectiveness in reducing polypharmacy and its consequent adverse outcomes is also a limitation of the tool at this stage, although an implementation study is underway.

### Strengths of the study

To the best of our `knowledge, this is the first deprescribing tool for older persons, developed in India, with inputs from over 24 experts in the above specialties. India is a large and diverse country. Therefore, experts from different states of India were involved, so that all regions were reasonably represented. Our tool includes important herb-drug interactions in addition to modern drugs. Also, unlike the most commonly used existing deprescribing tools, it suggests alternatives wherever possible, to be used in place of the drugs to be avoided.

### Conclusion and future direction

A deprescribing tool containing drugs and/or drug groups commonly prescribed and relevant for older persons in India has been developed through the Delphi process followed by tool validation. However, the tool has not yet been tested for clinical outcomes such as impact on reducing inappropriate polypharmacy or adverse events. A clinical effectiveness and feasibility study is being carried out as part of on-going work by the team, in two states of India- one each from the North (AIIMS Bhopal) and South (St. John’s Medical College, Bengaluru).

## Supplementary Information

Supplementary Material 1.

Supplementary Material 2.

## Data Availability

The data that support the findings of this study are not openly available due to reasons of confidentiality and are available from the corresponding author upon reasonable request. Data are located in controlled access data storage at All India Institute of Medical Sciences, Bhopal, India.

## Abbreviations

ACA: Anti-cholinergic action
AGS: American Society of Geriatrics
AIIMS: All India Institute of Medical Sciences
ASD: Anti-Psychotic Drugs
BZD: Benzodiazepine
CDSCO: Central Drugs Standard Control Organization
CVI: Content Validity Index
CVR: Content Validity Ratio
FDC: Fixed Dose Combination
HFrEF: Heart Failure with Reduced Ejection Fraction
ICMR: Indian Council of Medical Research
I-CVI: Item Level Content Validity Index
PIM: Potentially Inappropriate medications
S-CVI: Scale Level Content Validity Index
STOPP: Screening Tool for Older Person’s Prescriptions
