# Supplementary material for: Development and validation of a deprescribing tool relevant to older persons in India using modified Delphi consensus technique
Source: BMC Geriatr. 2025 Dec 2;25:991. doi: 10.1186/s12877-025-06665-3 (PMC12673781; doi:10.1186/s12877-025-06665-3)
Supplement: Supplementary file 1 — Supplementary Material 1. [file 12877_2025_6665_MOESM1_ESM.docx]

**Supplementary Table 1: Steering Committee Members, Delphi Panellists and Validation Experts**

| **Steering Committee Members** | | | |
| --- | --- | --- | --- |
| **S No.** | **Speciality** | **Expert** | **Affiliation** |
|  | Ayurveda, Yoga, Unani, Siddha and Homeopathy (AYUSH) | Dr. Danish Javed, Senior Medical Officer | AIIMS, Bhopal |
|  | Cardiology | Dr. Bhushan Shah, Asst. Professor | AIIMS, Bhopal |
|  | Clinical Pharmacology | Dr Ratinder Jhaj, Professor | AIIMS, Bhopal |
|  | Pharmacology | Dr. Atiya Faruqui, Professor | St Johns Medical College, Bengaluru |
|  | General Medicine | Dr Rajnish Joshi, Professor & Head | AIIMS, Bhopal |
|  | Geriatric Medicine | Dr Minakshi Dhar, Professor & Head | AIIMS Rishikesh |
|  | Psychiatry | Dr Snehil Gupta, Assoc. Professor | AIIMS, Bhopal |
|  | Orthopaedics | Dr. Rehan Ul-Haq, Professor & Head | AIIMS, Bhopal |
| **Delphi Panellists** | | | |
|  | Cardiology | Dr. Barun Kumar, Add. Professor, | AIIMS Rishikesh |
|  |  | Dr. Satyajeet Singh, Add. Professor & Head | AIIMS, Raipur |
|  |  | Dr. Kiron Varghese, Professor & Head | St. John's Medical College Hospital, Bengaluru |
|  | Clinical Pharmacology | Dr Santanu K Tripathi, Professor & Head | Calcutta School of Tropical Medicine, Kolkata |
|  |  | Dr. Ashish Kakkar, Professor | PGIMER Chandigarh |
|  |  | Dr. Sandhiya Selvarajan, Addl Prof & Head | JIPMER, Pondicherry |
|  | General Medicine | Dr Vishakha Jain, Add. Prof & Head | AIIMS Bibinagar |
|  |  | Dr Bidita Khandelwal, Professor | SMIMS Gangtok |
|  |  | Dr Ravi Kirti, Professor | AIIMS Patna |
|  | Geriatric Medicine | Dr Ashish Goel, Professor & Head | AIMS, Mohali, Punjab |
|  |  | Dr. Arunansu Talukdar, Professor & Head | Kolkata Medical College |
|  |  | Dr Vinod Joseph Abraham, Professor & Head | CMC Vellore |
|  | Orthopaedics | Dr Tarun Goyal, Professor & Head | AIIMS, Bhatinda |
|  |  | Dr Pankaj Kandwal, Professor & Head | AIIMS, Rishikesh |
|  |  | Dr. John A Santoshi, Professor | AIIMS, Bhopal |
|  | Psychiatry | Dr Nishant Goyal, Professor | CIP Ranchi |
|  |  | Dr PT Sivakumar, Professor & Head | NIMHANS, Bengaluru |
|  |  | Dr. Mathew Varghese, Senior Professor | St. John's Medical College Hospital, Bengaluru |
| **Tool Validation Experts** | | | |
|  | Cardiology | Dr. Jaideep Menon, Professor | Amrita Institute of Medical Sciences, Kochi |
|  | Clinical Pharmacology | Dr. SP Dhaneria, Prof. & HOD Pharmacology | RG Gardi Medical College, Ujjain |
|  | General Medicine | Jyotirmoy Pal, Professor | R G Kar Medical College & Hospital, Kolkata |
|  | Geriatric Medicine | Dr Arvind Mathur, Director and Managing Trustee | Asian Centre for Medical Education, Research & Innovation (ACMERI), Jodhpur |
|  | Psychiatry | Dr. Sandeep Grover | PGIMER, Chandigarh |
|  | Orthopaedics | Dr. Anil Jain, Indian Spine Journal  Editor-in-chief | Annals of National Academy of Medical Sciences, India |

**Supplementary Table 2: Deprescribing Tool for Older Persons Sent for Delphi Round 1**

Older persons (≥ 60 years) are often recipients of polypharmacy, exposing them to risks of adverse reactions and interactions. A review of medications that an older patient is taking needs to be carried out at every opportunity. This is a tool to aid medication review and identify medicines which may not be appropriate for an older patient. Only drugs with moderate or high quality of evidence and a strong strength of recommendation in published literature are included below. The advice below is only a recommendation and prescribers are in no way compelled to follow the same. Suggested alternatives *may have their own contraindications and interactions* which will need to be kept in mind. For complete information on drug interactions, please refer to online resources like https://www.drugs.com/professionals.html or scan the QR code(Will be provided in final tool)

.**Section I: Drugs which are considered inappropriate and should be generally avoided in an older patient**

| **S. No**. | **Drug/ Drug Group** | **Recommendation** | **Suggested Alternatives** | **Rationale & Reference** |
| --- | --- | --- | --- | --- |
| **Group A: Drugs with Anticholinergic action** | | Avoid *esp*. in dementia, delirium, narrow-angle glaucoma, constipation, prostatism & urinary retention, history of falls.  Avoid >1 drug with anticholinergic action. | | Anticholinergic action can cause cognitive decline, falls & constipation in older people. Higher cumulative use of anticholinergics associated with increased risk of dementia & mortality in an older person (1) |
|  | **First-Generation Antihistamines**  Chlorpheniramine, Clemastine, Cyproheptadine, Dexchlorpheniramine, Dimenhydrinate, Doxylamine, Hydroxyzine, Meclizine, Promethazine, Triprolidine | As for Group A.  *Appropriate* for treatment of severe allergic reaction | Second Generation Antihistamines e.g. Cetirizine, Levocetirizine, Fexofenadine,  Loratadine, Desloratadine etc. |  |
|  | **Antiparkinsonian agents** Benztropine, Trihexyphenidyl | As for Group A | Levodopa-carbidopa/benserazide. Dopamine agonists, COMT inhibitors or MAO Inhibitors if required.  Use minimum doses of all drugs (2) |  |
|  | **Antimuscarinics for urinary incontinence**  Darifenacin, Fesoterodine, Flavoxate, Oxybutynin , Solifenacin, Tolterodine, Trospium | Avoid. If necessary, use Trospium or Darifenacin | Behavioral therapy, Mirabegron (beta-3-agonist) if CrCl> 15 mL/min, no hepatic disease or uncontrolled hypertension and patient can afford. (3) | Trospium and Darifenacin have lower risk of cognitive effects (4) |
|  | **Gastrointestinal antispasmodics and anticholinergics**  Clidinium, Dicyclomine, Hyoscyamine, Propantheline, Scopolamine | Avoid, esp. for long term use. | Diet therapy (fiber, fluids); Loperamide, aluminum hydroxide, cholestyramine for diarrhea associated with Irritable Bowel Syndrome (5) |  |
|  | **Peripheral muscle relaxants**  Cyclobenzaprine, Carisoprodol, Chlorzoxazone, Metaxalone, Orphenadrine, Tizanidine | As for Group A.  If necessary, Tizanidine may be used at low doses in absence of liver disease. | Physiotherapy; correct seating & footwear; For acute mild or moderate pain—paracetamol ( < 3g/day) (6)  For spasticity- baclofen or nerve blocks (for localised spasticity)(5), (7) | Most muscle relaxants have anticholinergic adverse effects, sedation, increased risk of fractures.(8) |
|  | **Tricyclic Antidepressants with potent anticholinergic action**  Amitriptyline, Amoxapine, Clomipramine, Doxepin, Protriptyline, Trimipramine | Avoid.  Also avoid in syncope.  If necessary use Desipramine or Nortriptyline due to lower anticholinergic action.(5) | For depression—SSRI esp. with shorter half-life e.g. escitalopram, sertraline; SNRI, bupropion  For neuropathic pain—SNRI, capsaicin topical, gabapentin, pregabalin, lidocaine patch. (6) | Highly anticholinergic, sedating, & cause orthostatic hypotension.(8) |
|  | **Antipsychotic drugs (ASDs) with potent anticholinergic action**  **First Generation (FGA):** Chlorpromazine, Flupenthixol, Fluphenazine  **Second generation (SGA):**  Clozapine, Olanzapine | Avoid | **Antipsychotic drugs (ASDs) with less anticholinergic activity**-Haloperidol, Aripiprazole, Quetiapine, Risperidone | Increased risk of stroke & greater rate of cognitive decline & mortality in persons with dementia. (8)  FGA may worsen Parkinson’s.  Increased risk of thrombosis.(9) |
| 8 | **All Antipsychotic drugs (ASDs)** | Avoid for treatment of behavioural & psychological symptoms of dementia (BPSD). Avoid for > 12 weeks if history of coronary, cerebral or peripheral vascular disease  Avoid in Parkinson’s *except* Pimavanserin & quetiapine. | If necessary, use lowest effective dose of agents with less anticholinergic activity (see above) for shortest period of time.  Review at least every 6 weeks (10) |  |

| **Group B: Sedatives & Hypnotics** | | **Avoid esp. if history of falls.**  **Avoid > 3 drugs acting on the CNS** |  |  |
| --- | --- | --- | --- | --- |
| 1 | **Barbiturates** Phenobarbital | Avoid *except* for acute use in seizure disorders | For sleep— Sleep hygiene; cognitive behavioural therapy; minimise  use of caffeine and alcohol (6) | Cause more adverse effects than other sedatives/hypnotics in older persons, highly addictive(11). |
| 2 | **Benzodiazepines (BDZs)** **Short & intermediate acting:** Alprazolam, Estazolam, Lorazepam  Oxazepam, Temazepam, Triazolam  **Long acting:** Chlordiazepoxide, Clonazepam, Clorazepate Diazepam, Flurazepam, Quazepam | Avoid, *esp*. long acting BDZs,  *except* for use in seizure disorders, benzodiazepine withdrawal, ethanol withdrawal, severe generalized anxiety disorder & anaesthesia | For anxiety—buspirone, SSRI, SNRI  For sleep— Sleep hygiene; cognitive behavioural therapy; minimise use of caffeine and alcohol (6) | Increased sensitivity to benzodiazepines & decreased metabolism in older persons causing increased risk of cognitive impairment, delirium, falls, fractures & road traffic accidents. (8) |
| 3 | **Nonbenzodiazepine BDZ receptor agonist hypnotics (Z-drugs)** Eszopiclone, Zaleplon, Zolpidem | Avoid | Nonpharmacological therapy (6) | Adverse events similar to benzodiazepines in older adults & minimal improvement in sleep latency & duration. (8) |

| **Group C: Drugs acting on the Cardiovascular System & Blood** | | | | | |
| --- | --- | --- | --- | --- | --- |
| **S.**  **No**. | | **Drug/Drug Group** | **Recommendation** | **Suggested Alternatives** | **Rationale & Reference** |
| **Antithrombotic/Antiplatelet drugs** | | | | | |
| 1 | | Aspirin | Primary prevention of CVD: Avoid starting aspirin.  Consider deprescribing aspirin if already taking it.  Secondary prevention in CVD, reduce dose to < 100 mg /day (9) **HDI:** Gingko may increase bleeding time (12) ( see Table 2) | Diet & Lifestyle advice for primary prevention | No evidence of benefit. Risk of major bleeding from aspirin increases markedly in older age. (8), (13) |
| 2 | | Warfarin | Avoid as initial therapy for treatment of nonvalvular AF or VTE unless DOACs are contraindicated.  If already on long- term warfarin, may continue if INR well-controlled & no adverse effects.  Avoid, if possible, *with* drugs which affect coagulation or Warfarin action* **HDI:** Ginseng may ↓ INR, Garlic, Gingko may ↑INR (12), (14), (15) ( see Table 2) | DOACs esp. Apixaban, Edoxaban  *See Table 3 in case of renal disease* | Warfarin has higher risks of major bleeding (particularly intracranial bleeding) & similar or lower effectiveness for treatment of non-valvular AF or VTE as compared to DOACs. (8)  *(<https://www.drugs.com/professionals.html>) |
| 3 | | Rivaroxaban | Avoid for long-term treatment of AF or VTE.  Use only in special situations, e.g. when once-daily dosing is necessary to facilitate medication adherence.  Avoid if CrCl<15 mL/min. Reduce the dose if CrCl 15–50 mL/min | Apixaban, Edoxaban  *See Table 3 in case of renal disease* | Higher risk of major bleeding & GI bleeding in older adults than other DOACs, esp. apixaban.(8), (16) |
| **Cardiovascular Drugs** | | | | | |
| 4 | | Amiodarone | Avoid as first-line therapy for supraventricular tachyarrhythmias unless HF or substantial LVH (1), (8), (9) | Depends on type of arrhythmia | More adverse effects than other antiarrhythmics used in supraventricular tachyarrhythmias like beta-blockers, verapamil or diltiazem. (9) |
| 5 | | Dronedarone | Avoid ***in*** permanent AF or severe or recently decompensated HF. Use caution ***in*** patients with HFrEF with less severe symptoms (NYHA class I or II). | Depends on type of arrhythmia | Worse outcomes in people who have permanent AF or severe or recently decompensated HF. Worse outcomes have also been reported in NYHA class I or II HFrEF (LVH ≤35%) (8) |
| 6 | | Clonidine, Moxonidine | Avoid | ACEI/ARB, CCB, Thiazides.  Beta blocker if indicated | Centrally acting antihypertensives generally less well tolerated in older persons. High risk of CNS adverse effects, orthostatic hypotension and falls (3), (9), (17) |
| 7 | | Disopyramide | Avoid | AF: For rate control—Non DHP CCB e.g., diltiazem; beta-blocker  For rhythm control—dofetilide flecainide, propafenone; long-acting DHP CCB e.g., amlodipine.(6) | Disopyramide has anticholinergic properties and is known to worsen symptoms of prostatism, which is prevalent in older men. |
| 8 | | Digoxin | Avoid as first-line therapy for AF If used, avoid dosages >0.125 mg/day esp. if CrCl< 30ml/min | Depends on co-morbidities | Safer & more effective alternatives like beta- blockers available for rate control.(8),(9) |
| 9 | | Diltiazem, Verapamil | Avoid ***in*** Heart Failure  Avoid ***with*** Beta blockers* | Depends on indication | May worsen heart failure with reduced ejection fraction.  Increased risk of heart block with betablockers.(9) |
| 10 | | **Peripheral alpha-1 blockers** Alfuzosin, Doxazosin  Prazosin, Terazosin | Avoid esp. ***in*** syncope for lower urinary tract symptoms due to BPH. | Alpha-1A blockers: Silodosin, Tamsulosin (9) | High risk of orthostatic hypotension & falls, especially in older persons.(8) |
| 11 | | Statins | Avoid for primary cardiovascular prevention in > 75 years frail older persons | Diet & Lifestyle advice for primary prevention | For patients >75 years who are not frail, assessment of risk status and a clinician patient risk discussion are needed to decide whether to continue or initiate statin treatment.(18) |
| **Group D: Analgesics and Non- Steroidal Anti-inflammatory Medicines (NSAIMs)** | | | | | |
| 1 | | All Opioids | Avoid continuous use for > 3 months unless severe pain e.g. due to malignancy, and response to non-opioid or non-drug therapeutic interventions inadequate. | Depends on cause and severity of pain. | Opioids increase risk of falls and fractures in older persons (19) and dementia esp. in those 75- 80 years (20) |
| 2 | | Pethidine (Meperidine) | Avoid | Depends on cause and severity of pain. | Older persons are more sensitive to meperidine’s side effects including delirium. Decreased renal function may lead to accumulation of neurotoxic metabolite  ( normeperidine)(8),(21), (22) |
| 3 | | All NSAIDs | Avoid ***in*** Heart Failure, Renal disease with CrCl< 30 (mL/min)  Avoid ***with*** ACEI/ARB/ARNI, Diuretics, | Depends on cause and severity of pain.  Paracetamol (< 3g/day) for relief of pain or fever | Potential to promote fluid retention and/or  exacerbate heart and renal function decline.(8), (9) |
| 4 | | Indomethacin Ketorolac | Avoid |  | Increased risk of gastrointestinal bleeding/peptic ulcer disease & acute kidney injury in older adults. Indomethacin has higher risk of CNS effects. (8) |
| 5 | | **Non-COX-2-selective NSAIDs**  Aceclofenac, Aspirin* Diclofenac, Ibuprofen Indomethacin,  Ketorolac, Naproxen,Piroxicam | Avoid chronic use unless other alternatives are not effective.  Prescribe a PPI or misoprostol along with NSAID.  Avoid combination with systemic corticosteroids, anticoagulants, or antiplatelet agents unless other alternatives are not effective.  *Low-dose aspirin for antiplatelet action– see Group C |  | Increased risk of GI bleeding or peptic ulcer  disease esp. if >75 years old or taking oral or parenteral  corticosteroids, anticoagulants, or antiplatelet agents. Also, NSAIDs can increase blood pressure and induce kidney injury. (8), (9), (23) |
| 6 | | Serratiopeptidase  (alone or in combination) | Avoid | None | Insufficient evidence of efficacy and safety. (24), (25), (26) |
| **Group E: Gastrointestinal Drugs** | | | | | |
| 7 | **Proton-pump inhibitors**  Dexlansoprazole Esomeprazole  Lansoprazole Omeprazole  Pantoprazole Rabeprazole | | Avoid for >8 weeks unless for high-risk patients (e.g., oral corticosteroids or chronic NSAID use), erosive esophagitis, Barrett's esophagitis, pathologic hypersecretory condition, or failure of drug discontinuation trial or H2-receptor antagonists (H2RAs). | Behavioural & lifestyle management. As needed antacid, H2RA or PPI instead of daily PPI. Daily H2RA *except* Cimetidine. (27) | Risk of C. difficile infection, pneumonia, GI malignancies, bone loss, and fractures (8), kidney disease and dementia.(28), (29) H2RAs have lower risk of the above adverse effects. Cimetidine has to higher CNS and Endocrine adverse effects.(28) |
| 8 | Metoclopramide | | Avoid, unless for gastroparesis with a duration of use < 12 weeks. If used, prescribe lower starting dose of 5 mg four times daily. | Nausea/Vomiting- 5HT3 inhibitors e.g. Ondansetron (5)  Other drugs depending on indication | Can cause extrapyramidal effects, including tardive dyskinesia; the risk may be greater in older adults and with prolonged exposure and in Parkinson’s.(8), (30), (31) |
| 9 | Domperidone  (alone or in combination with PPIs) | | Avoid |  | Small increased risk of serious cardiac ADRs - QTc prolongation, torsade de pointes, serious ventricular arrhythmia & sudden cardiac death. A higher risk was observed in patients older than 60 year (32) |
| **Group F: Genitourinary Drugs** | | | | | |
| Desmopressin | | | Avoid for treatment of nocturia or nocturnal polyuria *esp.* *in* liver or renal disease and congestive heart failure. | Nonpharmacological management or drugs depending on cause | High risk of hyponatremia, especially in older persons (8), (9), (33). Risk higher in patients with liver or renal failure, or congestive heart failure. |

| **Group G: Drugs used in Endocrine disorders** | | | | |
| --- | --- | --- | --- | --- |
| 1 | **Sulfonylureas (SUs)**  **Long-acting:**  Glibenclamide (Glyburide), Glimepiride  **Short-acting**  Gliclazide, Glipizide | Avoid all SUs if possible.  If required, prefer short acting SUs  Avoid Glibenclamide & Glimepiride *esp***.** in Renal Disease.  **HDI:** Hypoglcemia may be exacerbated by Asian Ginseng (see Table2) | Metformin as first line. Other Antidiabetics depending on disease control, co-morbidities and patient acceptance | Long acting SUs: Higher risk of severe prolonged hypoglycaemia in older adults (8), (34)  Also, Glibenclamide & Glimepiride have active metabolites excreted by the kidney. Glipizide has inactive metabolites, hence preferred sulfonylurea in renal disease |
| 2 | Pioglitazone | Avoid if possible |  | Risk of edema and congestive heart failure. Increased bone loss and fracture risk esp. in older women (34), (35) |
| 3 | Insulin | Type 2 DM: Avoid insulin if possible. Use sparingly if necessary.  Type 1 & Type 2 DM: Avoid complex regimens and tight control of glucose. Monitor blood glucose frequently in addition to HbA1C |  | Higher risk of hypoglycaemia without improvement in hyperglycaemia management.(8), (34), (35). |
| 4 | Systemic estrogens with or without progestins for hormone replacement therapy | Avoid starting.  Consider deprescribing if already taking it. | Topical low-dose (estradiol<25 mcg twice weekly) vaginal cream or vaginal tablets  for dyspareunia, other vaginal symptoms & recurrent lower UTIs | Evidence of carcinogenic (breast and endometrium) potential & lack of cardiovascular and cognitive protection in women who start HRT after 60 years (36)  Risks of HRT (heart disease, stroke, blood clots, and dementia) more than benefits in women who start HRT after 60 years. (8)  Topical estrogens may be safe. |
| 5 | Megestrol for weight gain | Avoid | None | Minimal effect on weight; increases risk of thrombotic events & possibly death in older adults. (8), (37) |
| 6 | Growth hormone | Avoid, except for patients with definite diagnosis of growth hormone deficiency and established cause | None | Impact on body composition is small, no increase in stretch. Older persons are more sensitive to adverse effects like soft tissue edema, arthralgia, carpal tunnel syndrome, gynecomastia, impaired fasting glucose. (8),(38), (33) |
| 7 | Levothyroxine | Avoid for subclinical hypothyroidism | None | No evidence of benefit, risk of iatrogenic thyrotoxicosis (9) |

AF: Atrial Fibrillation; BB: Beta Blocker; BPH: Benign Prostatic Hypertrophy; CCB: Calcium Channel Blocker; CVD: Cardiovascular Disease; DOAC: Direct Oral Anti-coagulants; HDI: Herb-Drug Interaction; SSRI: Selective Serotonin Reuptake Inhibitors, SNRI: Serotonin Norepinephrine Reuptake Inhibitor; VTE: Venous Thromboembolism

**Section II: Commonly used herbal medicines with moderate level of drug interaction with allopathic medicines and recommendation to generally avoid giving them together** (39)

| **S No.** | **Allopathic medicine** | **Herbal medicine** | **Interaction** |
| --- | --- | --- | --- |
|  | Warfarin Aspirin, NSAIDS, dipyridamole, clopidogrel, prasugrel, ticagrelor | Ginkgo biloba, Allium sativum (Garlic, bulb/clove) | Increased bleeding due to synergism (15), (40) |
|  | Warfarin | Panax ginseng (Asian ginseng) | Decreased Warfarin effect due to Cytochrome P450 induction (14) |
|  | Insulin, Sulfonylureas, Glinides | Panax ginseng (Asian ginseng) | Hypoglycemia is a particular concern for older patients, and it can be exacerbated by ginseng.(41), (42) |
|  | Corticosteroids & various drugs | Licorice (Glycyrrhiza glabra)/Mulaithi | Licorice inhibits Cytochrome P450 enzymes affecting metabolism of a number of drugs. Check for interactions at https://www.drugs.com/drug-interactions/licorice-index.html |
|  | Furosemide | Panax ginseng (Asian ginseng) | Ginseng decreases the effect of furosemide, due to unknown mechanism. (41) |

**Section III:** Commonly used drugs which should be avoided in patients with renal dysfunction, due to risk of adverse effects. (8), (9), (43), (44)

Note: This is NOT a comprehensive list. For complete information on drug use and dose adjustments in renal dysfunction, please refer to online resources like https://www.drugs.com/professionals.html or scan the QR code (Will be provided in final tool)

| **S No.** | **Drugs which should be Avoided** | **Creatinine Clearance (mL/min)** | **Rationale** |
| --- | --- | --- | --- |
|  | Anticoagulants: Dabigatran, Fondaparinux | <30 | Risk of bleeding |
|  | Anticoagulants (Factor Xa inhibitors): apixaban, edoxaban, rivaroxaban | < 15 (Edoxaban: Reduce dose at 15-50; Rivaroxaban: VTE treatment & prophylaxis: Avoid at <30) |  |
|  | Bisphosphonates* | < 30 | Risk of acute renal failure |
|  | Digoxin | < 30 | Risk of digoxin toxicity |
|  | Diuretics (K sparing) ^†^ | <30 | Risk of hyperkalaemia |
|  | Duloxetine | <30 | Increased GI adverse effects |
|  | Dofetilide | <20: Avoid; 20-59: Reduce dose | Risk of QTc prolongation & torsades de pointes |
|  | Metformin | <30 | Risk of lactic acidosis |
|  | Methotrexate | <30 | Risk of methotrexate toxicity |
|  | NSAIDs | < 50 | Risk of deterioration in renal function |
|  | Pethidine (Meperidine) | <30 | Active metabolite, normeperidine, risk of seizure |
|  | Probenecid | <30 | Reduced efficacy |
|  | SGLT2 inhibitors^‡^ | < 30 | Reduced efficacy |
|  | Tramadol | <30 ER: Avoid; IR: reduce dose | Risk of tramadol toxicity |
|  | Trimethoprim sulfamethoxazole | <15: Avoid; 15-29: Reduce Dose | Risk of deterioration in renal function, hyperkalemia |
|  | **Herbal Drugs which should be Avoided** (43) |  |  |
|  | Licorice | <15 | Risk of sodium and water retention, hypokalemia, hypertension (43), (45), (46) |
|  | Noni juice | < 15 | Risk of hyperkalemia due to high potassium content (43), (46) |
|  | Ginkgo biloba | Avoid in patients with known  bleeding disorders and renal dysfunction | May improve renal function in early stages of Diabetic and Hypertensive nephropathy (47) (48). However, there is increased risk of bleeding and renal toxicity. (49) (43) |
|  | Ephedra alkaloids (ma huang) | Avoid in renal disease | Risk of nephrolithiasis (50) |

ER: Extended release, IR: Immediate release * Bisphosphonates: Alendronate, Ibandronate, Risedronate, Zoledronate etc.   ^†^ Diuretics (K sparing): Amiloride, Triamterene Spironolactone, eplerenone. ^‡^ SGLT2 inhibitors: Canagliflozin, Dapagliflozin, Empagliflozin etc

**REFERENCES**

1. Grossi CM, Richardson K, Savva GM, Fox C, Arthur A, Loke YK, et al. Increasing prevalence of anticholinergic medication use in older people in England over 20 years: cognitive function and ageing study I and II. BMC Geriatr. 2020 Dec;20(1):267.

2. Mouchaileh N, Hughes AJ. Pharmacological management of Parkinson’s disease in older people. J Pharm Pract Res. 2020 Oct;50(5):445–54.

3. Wang YC, Chen YL, Huang CC, Ho CH, Huang YT, Wu MP, et al. Cumulative use of therapeutic bladder anticholinergics and the risk of dementia in patients with lower urinary tract symptoms: a nationwide 12-year cohort study. BMC Geriatr. 2019 Dec;19(1):380.

4. López-Álvarez J, Sevilla-Llewellyn-Jones J, Agüera-Ortiz L. Anticholinergic Drugs in Geriatric Psychopharmacology. Front Neurosci. 2019 Dec 6;13:1309.

5. Potentially Harmful Drugs in the Elderly Beers List and More. Pharmacist’s Letter / Prescriber’s Letter 2007;23: 230907. Available from https://www.fmda.org/beers.pdf

6. Hanlon JT, Semla TP, Schmader KE. Alternative Medications for Medications in the Use of High‐Risk Medications in the Elderly and Potentially Harmful Drug–Disease Interactions in the Elderly Quality Measures. J Am Geriatr Soc [Internet]. 2015 Dec [cited 2024 May 14];63(12). Available from: https://agsjournals.onlinelibrary.wiley.com/doi/10.1111/jgs.13807

7. Trueman C, Castillo S, O’Brien KK, Pharm B, Hoie E. Inappropriate Use of Skeletal Muscle Relaxants in Geriatric Patients.

8. By the 2023 American Geriatrics Society Beers Criteria® Update Expert Panel. American Geriatrics Society 2023 updated AGS Beers Criteria® for potentially inappropriate medication use in older adults. J Am Geriatr Soc. 2023 Jul;71(7):2052–81.

9. O’Mahony D, Cherubini A, Guiteras AR, Denkinger M, Beuscart JB, Onder G, et al. STOPP/START criteria for potentially inappropriate prescribing in older people: version 3. Eur Geriatr Med. 2023 May 31;14(4):625–32.

10. NHS. Appropriate prescribing of antipsychotic medication in dementia. Available from https://www.england.nhs.uk/london/wp-content/uploads/sites/8/2022/10/Antipsychotic-Prescribing-Toolkit-for-Dementia.pdf 11. Leipzig RM. Avoiding adverse drug effects in elderly patients. Cleve Clin J Med. 1998 Oct 1;65(9):470–8.

12. Sheetal R M. Geriatrics. In: The Washington Manual of Medical Therapeutics. South Asian Edition adapted for local practices, conditions, and therapeutics. India: Wolters Kluwer Health, 2022. p. 1202-12

13. McNeil JJ, Wolfe R, Woods RL, Tonkin AM, Donnan GA, Nelson MR, et al. Effect of Aspirin on Cardiovascular Events and Bleeding in the Healthy Elderly. N Engl J Med. 2018 Oct 18;379(16):1509–18.

14. Dong H, Ma J, Li T, Xiao Y, Zheng N, Liu J, et al. Global deregulation of ginseng products may be a safety hazard to warfarin takers: solid evidence of ginseng-warfarin interaction. Sci Rep. 2017 Jul 19;7(1):5813.

15. K. Kapoor V, Singla S. Herb -Drug Interactions –An Update on Synergistic Interactions. J Altern Med Res [Internet]. 2015 Oct 5 [cited 2024 Feb 20];1(1). Available from: http://www.elynsgroup.com/journal/j-alt-med-res/article/herb-drug-interactions-an-update-on-synergistic-interactions

16. Silverio A, Di Maio M, Prota C, De Angelis E, Radano I, Citro R, et al. Safety and efficacy of non-vitamin K antagonist oral anticoagulants in elderly patients with atrial fibrillation: systematic review and meta-analysis of 22 studies and 440 281 patients. Eur Heart J - Cardiovasc Pharmacother. 2021 Apr 9;7(FI1):f20–9.

17. Welsh TJ, Mitchell A. Centrally acting antihypertensives and alpha-blockers in people at risk of falls: therapeutic dilemmas—a clinical review. Eur Geriatr Med. 2023 Jul 12;14(4):675–82.

18. Arnett DK, Blumenthal RS, Albert MA, Buroker AB, Goldberger ZD, Hahn EJ, et al. 2019 ACC/AHA Guideline on the Primary Prevention of Cardiovascular Disease: A Report of the American College of Cardiology/American Heart Association Task Force on Clinical Practice Guidelines. Circulation [Internet]. 2019 Sep 10 [cited 2024 Apr 29];140(11). Available from: https://www.ahajournals.org/doi/10.1161/CIR.0000000000000678

19. Virnes RE, Tiihonen M, Karttunen N, Van Poelgeest EP, Van Der Velde N, Hartikainen S. Opioids and Falls Risk in Older Adults: A Narrative Review. Drugs Aging. 2022 Mar;39(3):199–207.

20. Levine SZ, Rotstein A, Goldberg Y, Reichenberg A, Kodesh A. Opioid Exposure and the Risk of Dementia: A National Cohort Study. Am J Geriatr Psychiatry. 2023 May;31(5):315–23.

21. Swart LM, Van Der Zanden V, Spies PE, De Rooij SE, Van Munster BC. The Comparative Risk of Delirium with Different Opioids: A Systematic Review. Drugs Aging. 2017 Jun;34(6):437–43.

22. Friesen KJ, Falk J, Bugden S. The safety of meperidine prescribing in older adults: A longitudinal population-based study. BMC Geriatr. 2016 Dec;16(1):100.

23. Wongrakpanich S, Wongrakpanich A, Melhado K, Rangaswami J. A Comprehensive Review of Non-Steroidal Anti-Inflammatory Drug Use in The Elderly. Aging Dis. 2018;9(1):143.

24. Bhagat S, Agarwal M, Roy V. Serratiopeptidase: a systematic review of the existing evidence. Int J Surg. 2013;11(3):209–1717. Jadhav SB, Shah N, Rathi A, Rathi V, Rathi A. Serratiopeptidase: Insights into the therapeutic applications. Biotechnol Rep. 2020 Dec;28:e00544.

25. Jadhav SB, Shah N, Rathi A, Rathi V, Rathi A. Serratiopeptidase: Insights into the therapeutic applications. Biotechnol Rep. 2020 Dec;28:e00544.

26. Central Drug Standards Control Organisation (CDSCO). Report of the DTAB subcommittee appointed on 19.02.2018 to examine the matters related to 344 plus 5 FDCs in compliance with the Hon. Supreme court order dated 15.12.2017. Available from https://cdsco.gov.in/opencms/export/sites/CDSCO_WEB/Pdf-documents/Committee/dtab_sub_report.pdf

27. New South Wales Therapeutic Advisory Group. Deprescribing-Guide-for-Proton-Pump-Inhibitors-PPIs. Available from https://www.nswtag.org.au/wp-content/uploads/2018/06/1.9-Deprescribing-Guide-for-Proton-Pump-Inhibitors-PPIs.pdf

28. Maes ML, Fixen DR, Linnebur SA. Adverse effects of proton-pump inhibitor use in older adults: a review of the evidence. Ther Adv Drug Saf. 2017 Sep;8(9):273–97.

29. Dos Santos AS, De Menezes ST, Silva IR, Oliveira WN, Pereira ML, Mill JG, et al. Kidney function decline associated with proton pump inhibitors: results from the ELSA-Brasil cohort. BMC Nephrol. 2023 Sep 28;24(1):285.

30. Ghosn O, Ye E, Huege S. “Evaluating and Managing Tardive Dyskinesia in the Older Adult.” Curr Geriatr Rep. 2021 Sep;10(3):108–15.

31. FDA. Highlights Of Prescribing Information for metoclopramide (reglan). Available from https://www.accessdata.fda.gov/drugsatfda_docs/label/2017/017854s062lbl.pdf

32. European Medicines Agency. Restrictions on the use of domperidone-containing Restrictions on the use of domperidone-containing.pdf. Available from https://www.ema.europa.eu/en/documents/referral/domperidone-article-31-referral-restrictions--use-domperidone-containing-medicines_en.pdf

33. Fleseriu M, Hashim IA, Karavitaki N, Melmed S, Murad MH, Salvatori R, et al. Hormonal Replacement in Hypopituitarism in Adults: An Endocrine Society Clinical Practice Guideline. J Clin Endocrinol Metab. 2016 Nov 1;101(11):3888–921.

34. LeRoith D, Biessels GJ, Braithwaite SS, Casanueva FF, Draznin B, Halter JB, et al. Treatment of Diabetes in Older Adults: An Endocrine Society* Clinical Practice Guideline. J Clin Endocrinol Metab. 2019 May 1;104(5):1520–74.

35. Leung E, Wongrakpanich S, Munshi MN. Diabetes Management in the Elderly. Diabetes Spectr. 2018 Aug 1;31(3):245–53.

36. British Menopause Society. BMS & WHC’s 2020 recommendations on hormone replacement therapy in menopausal women. Available from https://thebms.org.uk/wp-content/uploads/2023/10/02-BMS-ConsensusStatement-BMS-WHC-2020-Recommendations-on-HRT-in-menopausal-women-SEPT2023-A.pdf

37. Persons RK, Nichols W, Papin KP. Clinical inquiries. Should we use appetite stimulants for malnourished elderly patients? J Fam Pract. 2007 Sep;56(9):761-2. PMID: 1776465129

38. Garcia JM, Merriam GR, Kargi AY. Growth Hormone in Aging. [Updated 2019 Oct 7]. In: Feingold KR, Anawalt B, Blackman MR, et al., editors. Endotext [Internet]. South Dartmouth (MA): MDText.com, Inc.; 2000-. Available from: https://www.ncbi.nlm.nih.gov/books/NBK279163/

39. Drugs.com. Drug Interaction Checker. Available from https://www.drugs.com/drug_interactions.html

40. Sharma AK, Kapoor VK, Kaur G. Herb–drug interactions: a mechanistic approach. Drug Chem Toxicol. 2022 Mar 4;45(2):594–603.

41. Charrois TL, Hrudey J, Vohra S. *Ginseng* : Practical Management of Adverse Effects and Drug Interactions. Can Pharm J Rev Pharm Can. 2006 Mar;139(2):44–6.

42. Brazier NC, Levine MAH. Drug-Herb Interaction Among Commonly Used Conventional Medicines: A Compendium for Health Care Professionals: Am J Ther. 2003 May;10(3):163–9.

43. Whittaker CF, Miklich MA, Patel RS, Fink JC. Medication Safety Principles and Practice in CKD. Clin J Am Soc Nephrol. 2018 Nov;13(11):1738–46.

44. Nespoux J, Vallon V. Renal effects of SGLT2 inhibitors: an update. Curr Opin Nephrol Hypertens. 2020 Mar;29(2):190–8.

45. Ceccuzzi G, Rapino A, Perna B, Costanzini A, Farinelli A, Fiorica I, et al. Liquorice Toxicity: A Comprehensive Narrative Review. Nutrients. 2023 Sep 5;15(18):3866.

46. Burrowes JD, Van Houten G. Use of Alternative Medicine by Patients With Stage 5 Chronic Kidney Disease. Adv Chronic Kidney Dis. 2005 Jul;12(3):312–25.

47. Zhang L, Mao W, Guo X, Wu Y, Li C, Lu Z, et al. Ginkgo biloba Extract for Patients with Early Diabetic Nephropathy: A Systematic Review. Evid-Based Complement Altern Med ECAM. 2013;2013:689142.

48. Jialiken D, Qian L, Ren S, Wu L, Xu J, Zou C. Combined therapy of hypertensive nephropathy with ginkgo leaf extract and dipyridamole injection and antihypertensive drugs. Medicine (Baltimore). 2021 May 14;100(19):e25852.

49. Li YY, Lu XY, Sun JL, Wang QQ, Zhang YD, Zhang JB, et al. Potential hepatic and renal toxicity induced by the biflavonoids from Ginkgo biloba. Chin J Nat Med. 2019 Sep;17(9):672–81.

50. Kiliś-Pstrusińska K, Wiela-Hojeńska A. Nephrotoxicity of Herbal Products in Europe—A Review of an Underestimated Problem. Int J Mol Sci. 2021 Jan;22(8):4132.
