# Supplementary material for: Development and validation of a deprescribing tool relevant to older persons in India using modified Delphi consensus technique
Source: BMC Geriatr. 2025 Dec 2;25:991. doi: 10.1186/s12877-025-06665-3 (PMC12673781; doi:10.1186/s12877-025-06665-3)
Supplement: Supplementary file 2 — Supplementary Material 2. [file 12877_2025_6665_MOESM2_ESM.docx]

**Table 2: Final Deprescribing Tool for older persons in India**

| 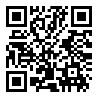**General Guidance on Drug Prescribing for older patients:** Older persons (≥ 60 years) are often recipients of polypharmacy, exposing them to risks of adverse reactions and interactions. This is a tool to aid medication review and identify medicines which may not be appropriate for an older patient. Only drugs with moderate or high quality of evidence and a strong strength of recommendation in published literature are included below. The advice below is only a recommendation & prescribers are in no way compelled to follow the same. Suggested alternatives *may have their own contraindications and interactions* which will need to be kept in mind. For complete information on drug interactions, please refer to online resources like https://www.drugs.com/professionals.html or scan the QR code.   - It may be appropriate to deprescribe a PIM in case of inappropriate indication, no current indication, presence or risk of adverse events,   adverse drug – drug or drug-disease interaction. ***Many drugs require the dose to be reduced slowly before stopping.***   - 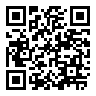Avoid irrational fixed dose combinations (FCDs) e.g. antimicrobials FDCs like fluoroquinolones plus nitroimidazoles, multimineral and multivitamin supplements. For complete list of all drugs including irrational FDCs banned in India please refer to <https://cdsco.gov.in/opencms/opencms/system/modules/CDSCO.WEB/elements/download_file_division.jsp?num_id=MTY4Mg==> or scan the QR code. - Also ask for history of medicines, including medicines from other systems of medicine. | | | | | | | |
| --- | --- | --- | --- | --- | --- | --- | --- |
| **Section I: Drugs which are considered inappropriate and should be generally avoided in an older patient** | | | | | | | |
| **S. No**. | **Drug/ Drug Group** | | **Recommendation** | | **Suggested Alternatives** | | **Rationale & Reference** |
| **Group A: Drugs with Anticholinergic action** | | | Avoid *esp*. in dementia, delirium, narrow-angle glaucoma, constipation, prostatism & urinary retention, history of falls.  **Avoid >1 drug with anticholinergic action**. | | | | Anticholinergic action can cause cognitive decline, falls & constipation in older people. Higher cumulative use of anticholinergics associated with increased risk of dementia & mortality in older persons (1) |
|  | **Tricyclic Antidepressants with potent anticholinergic action**  Amitriptyline, Amoxapine, Clomipramine, Protriptyline, Trimipramine | | As for all Group A Drugs.  Also avoid in syncope.  If TCA is necessary use Nortriptyline due to lower anticholinergic action (2) | | For depression—SSRIs, SNRI, bupropion  For neuropathic pain—SNRI, capsaicin topical, gabapentin, pregabalin, lidocaine patch (3) | | Highly anticholinergic, sedating, & cause orthostatic hypotension (4) |
|  | **Antipsychotic drugs (ASDs) with potent anticholinergic action**  **First Generation (FGA):** Chlorpromazine, Flupenthixol, Fluphenazine  **Second generation (SGA):**  Clozapine, Olanzapine | | Avoid.  Olanzapine or clozapine may have to be used if non-response to other drugs or risk of Extra-Pyramidal Symptoms (EPS).  Avoid all ASDs for treatment of behavioural & psychological symptoms of dementia (BPSD) for > 12 weeks, particularly if history of coronary, cerebral or peripheral vascular disease. | | **Antipsychotic drugs (ASDs) with less anticholinergic activity**-Haloperidol, Aripiprazole, Quetiapine, Risperidone  If necessary for BPSD, use lowest effective dose of agents with less anticholinergic activity for shortest period of time. Review at least every 6 weeks (5)  Prefer pimavanserin and quetiapine in Parkinson Disease. | | Increased risk of stroke & greater rate of cognitive decline & mortality in persons with dementia (4).  FGA may worsen Parkinson Disease.  Increased risk of thrombosis (6). |
|  | **Anticholinergic Antiparkinsonian agents** Benztropine, Trihexyphenidyl etc. | | As for all Group A Drugs | | Levodopa-carbidopa/benserazide. Dopamine agonists, COMT inhibitors or MAO Inhibitors if required. Use minimum doses of all drugs (7). | | As for all Group A Drugs |
|  | **Skeletal muscle relaxants**  Cyclobenzaprine, Carisoprodol, Chlorzoxazone, Metaxalone, Orphenadrine, Tizanidine | | As for all Group A Drugs.  If necessary, Tizanidine may be used at low doses in absence of liver disease. | | Physiotherapy; correct seating & footwear; For acute mild or moderate pain—paracetamol ( < 3g/day) (3). For spasticity- baclofen or nerve blocks (for localised spasticity) (2), (8) | | Most muscle relaxants have anticholinergic adverse effects, sedation, increased risk of fractures (4) |
|  | **Antimuscarinics for urinary incontinence**  Darifenacin, Fesoterodine, Flavoxate, Oxybutynin , Solifenacin, Tolterodine, Trospium | | Avoid.  If necessary, use Trospium or Darifenacin | | Behavioural therapy, Mirabegron if CrCl> 15 mL/min, no hepatic disease or uncontrolled hypertension & patient can afford (9) | | Trospium & Darifenacin have lower risk of cognitive effects (10) |
|  | **Gastrointestinal antispasmodics and anticholinergics**  Clidinium, Dicyclomine, Hyoscyamine, Propantheline, Scopolamine | | Avoid, esp. for long term use. | | Diet therapy (fibre, fluids); Medications may be used depending on cause of diarrhoea. | | As for all Group A Drugs |
|  | **First-Generation Antihistamines**  Chlorpheniramine, Clemastine, Cyproheptadine, Dexchlorpheniramine Dimenhydrinate, Doxylamine, Hydroxyzine, Meclizine, Promethazine, Triprolidine | | As for all Group A Drugs  *Appropriate* for treatment of severe allergic reaction | | Second Generation Antihistamines  e.g. Cetirizine, Levocetirizine,  Loratadine, Desloratadine etc. | | As for all Group A Drugs |
| **Group B: Sedatives & Hypnotics** | | | **Avoid esp. if history of falls. Avoid > 3 drugs acting on the CNS** | | | | |
|  | **Barbiturate**: Phenobarbital | | Avoid *except* for acute use in seizure disorders | | For sleep— Sleep hygiene; cognitive behavioural therapy; minimise use of caffeine and alcohol (3) | | Cause more adverse effects than other sedatives/hypnotics in older persons, highly addictive (11) |
|  | **Benzodiazepines (BZDs):** **Alprazolam,** Clonazepam, Diazepam, Flurazepam, Lorazepam, Oxazepam, Temazepam, Triazolam etc. | | Use the lowest possible dose, up to half of the usual dose.  Taper dose before stopping. | | For anxiety—buspirone, SSRI, SNRI  For sleep— Sleep hygiene; cognitive behavioural therapy; minimise use of caffeine and alcohol (3) | | Increased sensitivity to & reduced metabolism of BZDs in older persons cause increased risk of cognitive impairment, delirium, falls, fractures & road traffic accidents (4), (6), (12) |
| 3 | **Nonbenzodiazepine BZD receptor agonist hypnotics (Z-drugs)** Eszopiclone, Zaleplon, Zolpidem | | Avoid, if possible, esp. over 70 years. If necessary, use the lowest dose, up to half of usual dose and avoid for more than 2 weeks. In case of chronic insomnia may use up to 4-6 weeks, then taper and stop. | | Sleep hygiene; cognitive behavioural therapy; minimise use of caffeine and alcohol (3) If medication is necessary, melatonin up to 2 mg, one hour before bedtime may be used for the shortest duration necessary. | | Increased risk of falls & fractures when Z- drugs used for longer periods of time. (9), (13), (14). Melatonin may be a safe & effective option for older patients with insomnia, but long term safety is not established (15), (16). |
| **Group C: Drugs acting on the Cardiovascular System Disease (CVD) and Blood** | | | | | | | |
| **S.**  **No**. | **Drug/Drug Group** | **Recommendation** | | **Suggested Alternatives** | | **Rationale & Reference** | |
| **Antithrombotic/Antiplatelet drugs** | | | | | | | |
|  | Aspirin | Avoid starting aspirin for primary prevention of CVD. Consider stopping aspirin if already taking it.  For secondary prevention in CVD, reduce dose to less than 100 mg /day (6). Gingko biloba may increase bleeding time (See Section 2) | | Diet & Lifestyle advice for primary prevention | | No evidence of benefit for primary prevention of CVD. Risk of major bleeding from aspirin increases markedly in older age. (4), (17) | |
|  | Warfarin | Avoid starting therapy for treatment of nonvalvular AF or VTE unless DOACs are contraindicated.  If already taking warfarin, may continue if INR well-controlled & no adverse effects. See Section 2 for interactions with Ginseng, Garlic & Gingko | | DOACs esp. Apixaban, Edoxaban  *See Section III in case of renal disease* | | Warfarin has higher risks of major bleeding (particularly intracranial bleeding) & similar or lower effectiveness for treatment of non-valvular AF or VTE as compared to DOACs (4) | |
| **Cardiovascular Drugs** | | | | | | | |
|  | Centrally acting antihypertensives: Clonidine, Moxonidine | Avoid | | ACEI/ARB, CCB, Thiazides.  Beta blocker if indicated | | Centrally acting antihypertensives generally less well tolerated in older persons. High risk of CNS adverse effects, orthostatic hypotension and falls (3), (6), (18) | |
|  | Digoxin | Avoid as first-line therapy for AF If used, avoid dosages >0.125 mg/day esp. if CrCl< 30ml/min | | Depends on co-morbidities | | Safer & more effective alternatives like beta- blockers available for rate control (4),(6). | |
|  | Disopyramide | Avoid | | AF: For rate control—Non DHP CCB e.g., diltiazem; beta-blocker.  For rhythm control—dofetilide flecainide, propafenone; long-acting DHP CCB e.g. amlodipine (3) | | Disopyramide has anticholinergic properties and is known to worsen symptoms of prostatism, which is prevalent in older men. | |
|  | **Non- Dihydropidine CCBs:** Diltiazem, Verapamil | Avoid ***in*** Heart Failure  Avoid ***with*** beta blockers | | Depends on indication | | May worsen heart failure with reduced ejection fraction.  Increased risk of heart block with beta blockers.(6) | |
|  | **Peripheral alpha-1 blockers:** Alfuzosin, Doxazosin  Prazosin, Terazosin Tamsulosin | Avoid esp. ***in*** syncope, for lower urinary tract symptoms due to BPH. | | Alpha-1a blockers: Silodosin (6) | | High risk of orthostatic hypotension & falls, especially in older persons (4). | |

| **Group D: Analgesics and Non- Steroidal Anti-inflammatory Drugs (NSAIDs)** | | | | | | | | | | | | |
| --- | --- | --- | --- | --- | --- | --- | --- | --- | --- | --- | --- | --- |
|  | All Opioids | | | | | Avoid continuous use for > 3 months unless severe pain e.g. due to malignancy, & response to non-opioid or non-drug interventions inadequate. | | | | Depends on cause and severity of pain. | | Opioids increase risk of falls and fractures in older persons (19) and dementia esp. in those 75- 80 years (20). |
|  | Pethidine (Meperidine) | | | | | Avoid | | | | Depends on cause and severity of pain. | | Oder persons are more sensitive to CNS side effects of meperidine - anxiety, hallucinations, confusion, seizures. Also, renal function generally decreases with age, & normeperidine, a neurotoxic metabolite of pethidine may accumulate and cause seizures (4),(21), (22) |
|  | All NSAIDs | | | | | Avoid ***in*** Heart Failure and renal disease with CrCl less than 30 (mL/min) | | | | Paracetamol (less than 3g/day) for relief of pain or fever | | Potential to promote fluid retention & exacerbate heart & renal function decline (4), (6). May reduce effect pharmacological effects of ACEI/ARB/ARNI, Diuretics in heart failure & hypertension. |
|  | Indomethacin | | | | | Avoid | | | |  |  | Increased risk of gastrointestinal bleeding/peptic ulcer disease, acute kidney injury & CNS adverse effects in older persons (4), (23) |
|  | **Non-COX-2-selective NSAIDs**  Aceclofenac, Aspirin* Diclofenac, Ibuprofen Indomethacin,  Ketorolac, Naproxen, Piroxicam | | | | | Avoid chronic use unless other alternatives are not effective.  Prescribe a PPI or misoprostol along with NSAID.  Avoid combination with systemic corticosteroids, anticoagulants, or antiplatelet agents unless other alternatives are not effective.  *Low-dose aspirin for antiplatelet action– see Group C | | | |  |  | Increased risk of GI bleeding or peptic ulcer  disease esp. if >75 years old or taking oral or parenteral  corticosteroids, anticoagulants, or antiplatelet  agents. Also, NSAIDs can increase blood pressure and induce kidney injury. (4), (6), (24) |
| **Group E: Gastrointestinal** | | | | | | | | | | | | |
|  | | **Proton-pump inhibitors**  Esomeprazole  Lansoprazole Omeprazole  Pantoprazole Rabeprazole etc. | | | Avoid for >8 weeks unless for high-risk patients (e.g., oral corticosteroids or chronic NSAID use), erosive esophagitis, Barrett's esophagitis, pathologic hypersecretory condition.  May be appropriate if inadequate response with H2-receptor antagonists (H2RAs) or stopping of PPIs was not tolerated by patient. | | | | | | Behavioural & lifestyle management. As needed antacid, H2RA or PPI instead of daily PPI. Daily H2RA *except* Cimetidine. (25) | Risk of *C. difficile* infection, pneumonia, GI malignancies, bone loss, and fractures (4), kidney disease and dementia (26), (27).  H2RAs have lower risk of the above adverse effects.  Cimetidine has higher CNS and endocrine adverse effects (26) |
|  | | Metoclopramide | | | Avoid.  May use for gastroparesis at lower starting dose of 5 mg four times daily & for < 12 weeks | | | | | | Nausea/Vomiting- 5HT3 inhibitors e.g. Ondansetron (2)  Other drugs depending on indication | Older persons have higher risk of extrapyramidal effects, including tardive dyskinesia, especially with prolonged exposure and in Parkinson’s.(4), (28), (29) |
|  | | Domperidone | | | Avoid alone as well in combination with PPIs | | | | | |  | Older persons have slightly higher risk of serious cardiac ADRs - QTc prolongation, torsade de pointes, ventricular arrhythmia & sudden cardiac death (30). |
| **Group F: Genitourinary** | | | | | | | | | | | | |
| Desmopressin | | | | | Avoid for treatment of nocturia or nocturnal polyuria esp. in liver or renal disease and congestive heart failure | | | | | | Nonpharmacological management or drugs depending on cause | High risk of hyponatremia, especially in older persons (4), (6), (31). Risk higher in patients with liver or renal failure, or congestive heart failure. |
| **Group G: Drugs used in Endocrine disorders** | | | | | | | | | |  |  |  |
| 1 | **Sulfonylureas (SUs)**  **Long-acting:**  Glibenclamide (Glyburide), Glimepiride  **Short-acting**  Gliclazide Glipizide | | | Avoid, esp. avoid long-acting SUs in renal disease.  If required, prefer short acting Sus.  Hypoglycaemia may be exacerbated by Asian Ginseng (see Section 2) | Use metformin as first line. Use other Antidiabetics depending on disease control, co-morbidities and patient acceptance | Older persons are more prone to prolonged hypoglycaemia with long acting SUs (4), (32). Also, renal function generally decreases with age, leading to accumulation of active metabolites of glibenclamide & glimepiride. Gliclazide & glipizide have shorter duration of action and inactive metabolites, hence preferred if SU has to be used in Older persons. | | | |  |  |  |
| 2 | Pioglitazone | | | Avoid if possible |  | Risk of edema and congestive heart failure. Increased bone loss and fracture risk esp. in older women (32), (33) | | | |  |  |  |
| 3 | Systemic estrogens for hormone replacement therapy | | | Avoid starting (with or without progestins).  Consider stopping if already taking it. | Topical low-dose (estradiol<25 mcg twice weekly) vaginal cream or vaginal tablets for dyspareunia, other vaginal symptoms & recurrent lower UTIs | Evidence of carcinogenic (breast and endometrium) potential & lack of cardiovascular and cognitive protection in women who start HRT after 60 years (34)  Risks of HRT (heart disease, stroke, blood clots, and dementia) more than benefits in women who start HRT after 60 years. (4)  Topical estrogens may be safe. | | | |  |  |  |
| 4 | Levothyroxine | | | Avoid for subclinical hypothyroidism unless serum TSH persistently ≥10 mU/L | None | Older persons with TSH > 10 mU/L have increased incidence & prevalence of HF and those with preexisting HF have a poor prognosis (35). However, there may be no benefit & increased risk of iatrogenic thyrotoxicosis with levothyroxine when TSH ≤ 10 mU/L (6), (36), (37). | | | |  |  |  |

AF: Atrial Fibrillation; BB: Beta Blocker; BPH: Benign Prostatic Hypertrophy; CCB: Calcium Channel Blocker; CVD: Cardiovascular Disease; DOAC: Direct Oral Anti-coagulants; HDI: Herb-Drug Interaction; HF: heart Failure; PPI: Proton Pump Inhibitor; SSRI: Selective Serotonin Reuptake Inhibitors; SNRI: Serotonin Norepinephrine Reuptake Inhibitor; TSH: Thyroid Stimulating Hormone; VTE: Venous Thromboembolism

**Section II: Commonly used herbal medicines with moderate level of drug interaction with allopathic medicines and recommendation to generally avoid giving them together** (38)

| **S No.** |  | **Allopathic Medicine** | **Herbal Medicine** | **Interaction** |
| --- | --- | --- | --- | --- |
|  |  | Warfarin Aspirin, NSAIDS, dipyridamole, clopidogrel, prasugrel, ticagrelor | Ginkgo biloba, Allium sativum (Garlic, bulb/clove) | Increased bleeding due to synergism (39), (40), (41) |
|  |  | Warfarin | *Panax ginseng*  (Asian/Korean ginseng) | Decreased Warfarin effect due to Cytochrome P450 induction (41),(42), (43) |
|  |  | Insulin, Sulfonylureas, Glinides |  | Hypoglycemia is a particular concern for older patients, and it can be exacerbated by ginseng. (41), (43) (44), (45) |
|  |  | Furosemide |  | Ginseng decreases effect of furosemide, due to unknown mechanism. Until further data are available, patients on furosemide or other loop diuretics should preferably avoid use of ginseng supplements. Possibility of diuretic resistance should be considered and appropriately monitored if concomitant use is necessary. (38) |
|  |  | Potassium sparing diuretics, ACEIs, ARBs, Beta blockers | Noni juice  [Contains Noni Extract (*Morindo Citrifolia*), may contain *Garcinia Combojia* & other herbals] | Risk of hyperkalemia due to high K content in Noni Juice. (41). Increased risk in patients with renal disease (46). *Garcinia Combojia* may lead to liver injury, with and interact with other hepatotoxic drugs (47). |
|  |  | Corticosteroids & various drugs | Licorice (*Glycyrrhiza glabra)*/Mulaithi | Licorice inhibits Cytochrome P450 enzymes affecting metabolism of a number of drugs. Check for interactions at https://www.drugs.com/drug-interactions/licorice-index.html |
|  |  | Antihypertensives, including diuretics |  | Risk of sodium and water retention, hypokalemia, hypertension. (48) (49). Increased risk in patients with renal disease. (46) |


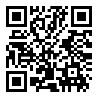
**Section** **III:** Commonly used drugs which should be avoided in patients with renal dysfunction, due to risk of adverse effects. (4), (6), (50)

Note: This is NOT a comprehensive list. For complete information on drug use and dose adjustments in renal dysfunction, please refer to online resources like https://www.drugs.com/professionals.html or scan the **QR code**

| **S No.** | **Drugs which should be Avoided** | **Creatinine Clearance (mL/min)** | **Rationale** |
| --- | --- | --- | --- |
|  | Anticoagulants: Dabigatran, Fondaparinux | <30 | Risk of bleeding (4), (6) |
|  | Anticoagulants (Factor Xa inhibitors): apixaban, edoxaban, rivaroxaban | < 15 (Edoxaban: Reduce dose at 15-50; Rivaroxaban: VTE treatment & prophylaxis: Avoid at <30) |  |
|  | Bisphosphonates* | < 30 | Risk of acute renal failure (6) |
|  | Digoxin | < 30 | Risk of digoxin toxicity (9) |
|  | Diuretics (K sparing) ^†^ | <30 | Risk of hyperkalemia (4), (6) |
|  | Duloxetine | <30 | Increased GI adverse effects, risk of liver toxicity (4), (51) |
|  | Dofetilide | <20: Avoid; 20-59: Reduce dose | Risk of QTc prolongation & torsades de pointes (4) |
|  | Metformin | <30 | Risk of lactic acidosis (6) |
|  | Methotrexate | <30 | Risk of methotrexate toxicity (6) |
|  | NSAIDs | < 30 | Risk of deterioration in renal function (4), (52) |
|  | Pethidine (Meperidine) | <30 | Active metabolite, normeperidine, risk of seizure (23) |
|  | Probenecid | <30 | Reduced efficacy (4) |
|  | SGLT2 inhibitors^‡^ | < 30 | Reduced efficacy (53) |
|  | Tramadol | <30 ER: Avoid; IR: reduce dose | Risk of tramadol toxicity (8) |
|  | Trimethoprim sulfamethoxazole | <15: Avoid; 15-29: Reduce Dose | Risk of deterioration in renal function, hyperkalemia (4) |

ER: Extended release, IR: Immediate release * Bisphosphonates: Alendronate, Ibandronate, Risedronate, Zoledronate etc.   ^†^ Diuretics (K sparing): Amiloride, Triamterene Spironolactone, eplerenone. ^‡^ SGLT2 inhibitors: Canagliflozin, Dapagliflozin, Empagliflozin etc.

**FOR REFERENCES, PLEASE SCAN THE QR CODE**

**
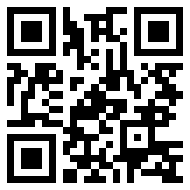
**
